# Molecular and morphological evidence of hybridization between two dimorphic sympatric species of *Fuchsia* (Onagraceae)

**DOI:** 10.1093/aobpla/plad089

**Published:** 2023-12-21

**Authors:** Cinthya Indira Cervantes-Díaz, Violeta Patiño-Conde, Antonio González-Rodríguez, Mauricio Quesada, Eduardo Cuevas

**Affiliations:** Laboratorio Nacional de Análisis y Síntesis Ecológica (LANASE), Escuela Nacional de Estudios Superiores, Unidad Morelia, Universidad Nacional Autónoma de México, Morelia 58190, Michoacán, México; Facultad de Biología, Universidad Michoacana de San Nicolás de Hidalgo 58000, Morelia, Michoacán, México; Laboratorio Nacional de Análisis y Síntesis Ecológica (LANASE), Escuela Nacional de Estudios Superiores, Unidad Morelia, Universidad Nacional Autónoma de México, Morelia 58190, Michoacán, México; Instituto de Investigaciones en Ecosistemas y Sustentabilidad, Universidad Nacional Autónoma de México, Morelia 58190, Michoacán, México; Laboratorio Nacional de Análisis y Síntesis Ecológica (LANASE), Escuela Nacional de Estudios Superiores, Unidad Morelia, Universidad Nacional Autónoma de México, Morelia 58190, Michoacán, México; Instituto de Investigaciones en Ecosistemas y Sustentabilidad, Universidad Nacional Autónoma de México, Morelia 58190, Michoacán, México; Laboratorio Nacional de Análisis y Síntesis Ecológica (LANASE), Escuela Nacional de Estudios Superiores, Unidad Morelia, Universidad Nacional Autónoma de México, Morelia 58190, Michoacán, México; Facultad de Biología, Universidad Michoacana de San Nicolás de Hidalgo 58000, Morelia, Michoacán, México

**Keywords:** chloroplast DNA sequences, *Fuchsia*, geometric morphometrics, hybridization, internal transcriber spacer (ITS), microsatellites

## Abstract

Hybridization is commonly reported in angiosperms, generally based on morphology, and in few cases confirmed by molecular markers. *Fuchsia* has a long tradition of ornamental cultivars with different hybrids produced by artificial crosses, so natural hybridization between sympatric *Fuchsia* species could be common. Natural hybridization between *F. microphylla* and *F. thymifolia* was tested using six newly developed microsatellites for *F. microphylla* in addition to other molecular markers with codominant and maternal inheritance. Geometric morphometrics of leaves and floral structures were also used to identify putative hybrids. Hybrids showed a different degree of genetic admixture between both parental species. Chloroplast DNA (cpDNA) sequences indicated that hybridization occurs in both directions, in fact, some of the hybrids showed new haplotypes for cpDNA and ITS (internal transcriber spacer of nuclear ribosomal RNA genes) sequences. The morphology of hybrid individuals varied between the two parental species, but they could be better identified by their leaves and floral tubes. Our study is the first to confirm the hybridization in natural populations of *Fuchsia* species and suggests that hybridization has probably occurred repeatedly throughout the entire distribution of the species. Phylogeographic analysis of both species will be essential to understanding the impact of hybridization throughout their complete distribution.

## Teaser

*Fuchsia* is a genus widely used for ornamental purposes, with different hybrids produced by artificial crosses. We confirmed for the first time the hybridization in natural populations of this genus, using molecular markers and geometric morphometrics of leaves and floral structures. The results suggest that hybridization has probably occurred repeatedly throughout the entire distribution of the species and hybridization occurs in both directions with respect to parental species. The morphology of hybrid individuals varied between the two parental species, but they could be better identified by their leaves and floral tubes.

## Introduction

Defining a species has always been a subject of debate (Aldhebiani 2018), especially for plants due to the frequent reports of hybridization and the enormous phenotypic variation exhibited by many groups of plants (Rieseberg and Willis 2007). The most used definition of species corresponds to the biological species concept as ‘groups of interbreeding organisms that can successfully interbreed and produce fertile offspring and are reproductively isolated from other groups of organisms’ (Mayr 1942; Aldhebiani 2018; Gustafsson *et al.* 2022). Reproductive isolation is a process that prevents individuals from different populations to mate and produce fertile offspring through pre-zygotic or post-zygotic barriers. Together these barriers are critical for driving speciation and maintaining species identity (Ramsey *et al.* 2003; Ouyang and Zhang 2018). Species that co-flower, share pollinators, and have incomplete reproductive barriers, may transfer interspecific pollen facilitating natural hybridization. This means that divergent lineages may meet, reproduce, and form at least some offspring of mixed ancestry (Keller *et al.* 2020). Hybridization may occur in different spatial and temporal contexts, for example, secondary contact after a period of independent evolution or primary intergradation with divergent selection, both cases occurring before species are fully reproductively isolated from each other (Abbott *et al.* 2013; Abbott 2017).

The initial recognition of hybrids can arise in different ways such as (i) artificial hybridization of cultivated plants, (ii) spontaneous hybridization of plants in gardens, (iii) from plants that had previously been regarded as species, or as infraspecific variants that were later recognized as hybrids and finally, (iv) some hybrids had been discovered by taxonomic specialists that subjected a genus to more detailed and critical study than they had hitherto received (Preston and Pearman 2015). The use of molecular markers helps to know the evolutionary consequences of hybridization which will always depend on its frequency and extent, as well as on the fitness of hybrids (Rieseberg *et al.* 1993; Pinheiro *et al.* 2010; Whitney *et al.* 2010). When the F_1_ hybrids are not reproductively isolated from their parents, they can promote introgression by recurrent backcrossing (Burgess and Husband 2004; Kirk *et al.* 2005; Field *et al.* 2009; Kamiya *et al.* 2011; Abbott 2017). In those cases, when reproductive isolation is incomplete and introgressive hybridization occurs, complex hybrid zones may be produced (Rhymer and Simberloff 1996; Abbott 2017).

Natural hybridization is a common phenomenon in plants and reports of spontaneous hybridization ranges from 25% at the species level to 40% at the family level; however, hybridization is not uniformly distributed among plant taxa (Ellstrand *et al.* 1996; Mallet 2007; Whitney *et al.* 2010).

Onagraceae stands out as one of the families with the highest propensity to hybridization; nonetheless, research with molecular markers in this family remains relatively limited (Whitney *et al*. 2010). From the 22 genera of the Onagraceae family, natural hybridization has been reported in species of *Ludwigia*, *Epilobium, Clarkia, Oenothera,* and *Fuchsia*; the first three genera include cases of polyploid species with the probable hybrid origin, while *Oenothera* has evolved a specialized system of permanent translocation heterozygosity (PTH), apparently as a product of hybridization. Finally, the success of artificial crosses in species of *Epilobium* and *Fuchsia*, suggests the possibility that natural hybridization is taking place (Wagner *et al.* 2007).

For *Fuchsia* (Onagraceae), a genus with over 110 species of mesic shrubs confined to cool, moist habitats, the phylogeny indicates a rapid initial diversification, due to its poor basal resolution, whose modern lineages are estimated to have diverged 31 mya (Berry *et al.* 2004). *Fuchsia* is well known for its ornamental cultivars developed from artificial crosses of some species of section *Quelusia* and *Ellobium* (Wagner *et al.* 2007; Talluri 2012). In addition, artificial hybridization has been performed between several species and different cultivars of *Fuchsia*, observing that the success of the crosses depends on the taxonomic distance between the species, indicating weak reproductive barriers (Talluri 2009, 2012; Talluri and Murray 2014). Natural hybridization has also been suggested among sympatric species of *Fuchsia* (Breedlove 1969; Talluri 2009). For example, *F. colensoi* was first described as a species but later it was discovered that *F. colensoi* individuals can be produced by artificial crosses between *F. excorticata* and *F. perscandens*. Therefore, *Fuchsia colensoi* changed its name to *F. x colensoi* although no differences were found in the chloroplast genome among the three species, which suggests a high level of introgression (Sytsma *et al.* 1991; Godley and Berry 1995). Natural hybridization between *F. regia* and several other sympatric *Fuchsia* species has also been suggested based on the apparent intermediate morphology (Berry 1989), but artificial crosses between *F. regia* and *F. campos-portoi,* did not produce fruits (Archer *et al.* 2021).In a similar way, the hybridization between *F. microphylla* and *F. thymifolia*, two closely related species from the section *Encliandra* (Berry *et al*. 2004) has been suggested (Breedlove 1969; Talluri 2011) but there is no evidence to demonstrate their hybridization in natural populations.

Here, we tested the hypothesis that individuals with intermediate morphology, found in a sympatric population of *F. microphylla* and *F. thymifolia* are hybrids. We used geometric morphometry and molecular markers to answer the following questions: Are *F. microphylla* and *F. thymifolia* hybridizing in a sympatric population? if so, what is the direction of hybridization? and finally, how is the morphology of the intermediate individuals compared to their parents?

## Materials and Methods

### Study species

*F. microphylla* and *F. thymifolia* are two morphologically gynodioecious but functionally subdioecious species of shrubs with female and hermaphrodite individuals, but hermaphrodite individuals function as males (Arroyo and Raven 1975; Cuevas *et al.* 2014). Both species occupy very similar habitats and almost the same range of distribution in Mexico, but *F. microphylla* extends to Panama; while *F. thymifolia* only reaches Guatemala (Breedlove 1969). The two studied species have sympatric and allopatric populations throughout their distribution and share some relevant features: hermaphrodite flowers are larger than female flowers, each flower has four petals and four sepals, as well as a tetra lobed stigma, a cylindrical or obconical floral tube, with eight stamens or staminodes in hermaphrodites and females, respectively. The flowers of *F. microphylla* are always purplish-red, while the petals of *F. thymifolia* are white when the flower opens and then turn purplish (two or three days after, Cervantes and Cuevas 2023). The leaves of *F. microphylla* are serrate from the apex to the middle, and those of *F. thymifolia* are sub entire. The literature reports different pollinators for each species (i.e. hymenopterans for *F. microphylla* and dipterans for *F. thymifolia*, Breedlove 1969; Arroyo and Raven 1975), but recent observations suggested that they may share at least one pollinator (*Bombus ephippiatus*) in the sympatric population of Garnica where some overlap in the flowering season has been observed (Cuevas *et al.* 2014; Cervantes *et al.* 2018).

### Study area

**Figure 1.**
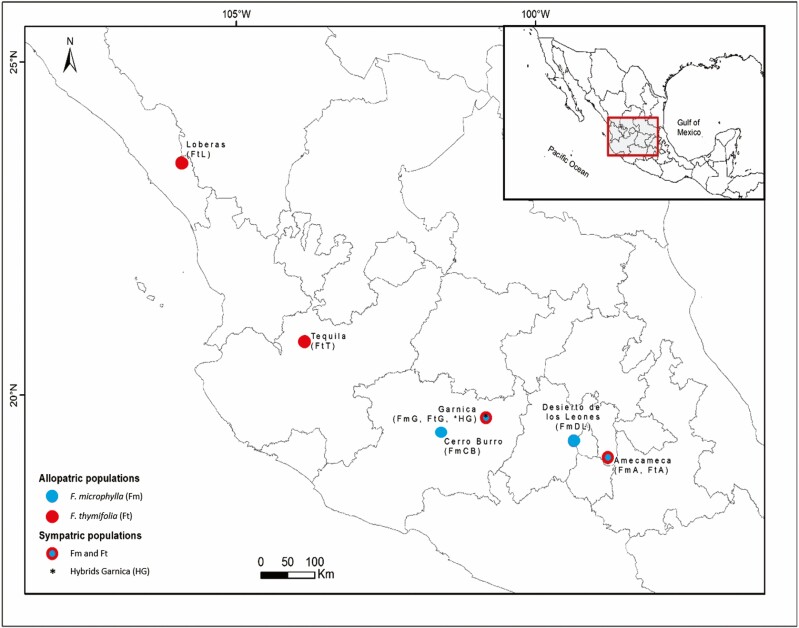
Map showing the location of sampled populations of *Fuchsia microphylla* and *F. thymifolia* in the Transmexican volcanic belt. The red dot shows the allopatric populations of *F. thymifolia*, and the blue dots the allopatric populations of *F. microphylla.* Sympatric populations are represented by two colors (blue and red). The putative hybrids were collected in the Garnica population indicated with an asterisk. The acronyms used for each species and population are indicated in parentheses.

The studied populations are located mainly in the Trans-Mexican Volcanic Belt (Fig. 1). In general, the climate of these mountains is temperate with rainfalls in summer with a mean annual temperature between 10 and 20 °C and between 400 and 1000 mm of annual precipitation (Hernández and Carrasco 2007). In general, both species are found on slopes, ravines, and humid areas of *Pinus, Abies, Quercus,* and mesophilic forests at elevations ranging from 2500 to 3400 m a.s.l. (Breedlove 1969; Arroyo and Raven 1975; Rzedowski *et al.* 2005).

Leaf tissue was collected from 212 individuals belonging to two allopatric populations of each species and two sympatric populations (Fig. 1; Table 1). Twenty to 32 individuals per species/per population, separated at least 3 m from each other, were randomly collected. The leaves were stored at −72°C or dried in silica until processed. Given the limited number of flowers during the leaves collection for the genetic analyses, the flowers, and leaves for the morphometric analyses were collected in different flowering seasons from different individuals and a smaller number of populations (one sympatric and one allopatric for each species; Table 1).

**Table 1.** Details of the sampled populations and the acronyms used. The code refers to the species name: Fm = *F. microphylla*, Ft = *F. thymifolia* and H = Hybrids. N = Number of individuals used for each genetic marker and geometric morphometrics. nSSR = nuclear microsatellites, cpDNA = chloroplast sequences, rnDNA = nuclear sequences from ITS, GM = geometric morphometrics.

Populations	Code	nSSR (*N*)	cpADN (*N*)	rnDNA (*N*)	GM (*N*)
Desierto de los Leones (DL)	FmDL	30	4	3	20
Cerro Burro (CB)	FmCB	30	3	3	
Amecameca (A)	FmA	20	3	3	
	FtA	20	3	3	
Garnica (G)	FmG	20	4	4	20
	FtG	20	4	4	20
	HG	32	32	13	30
Tequila (T)	FtT	20	4	2	20
Loberas (L)	FtL	20	3	3	
Total		212	60	38	110

In the sympatric population of Garnica, the individuals of each species were collected at random, while 32 individuals with intermediate morphology between *F. microphylla* and *F. thymifolia* were collected and considered as putative hybrids. The sympatric population of Amecameca was included in the study because Breedlove (1969) reported one hybrid in this population. However, at the time of collection there were no flowers to try to identify the hybrids, so the collection of leaves was at random. Allopatric populations for each species were included as a reference since it is likely that ‘pure’ individuals may not be found in sympatric populations due to possible hybridization.

### Nuclear simple sequence repeats (nSSRs)

Simple sequences repeats (SSR) were characterized *de novo* by Genetic Marker Services (Brighton, United Kingdom; www.geneticmarkerservices.com, see protocol and characterization of 15 loci from *F. microphylla* in the Supporting Information 1 (SI1)).

From the 15 developed loci, only seven were scorable and polymorphic for 40 individuals from *F. microphylla* and then were further tested using the PCR conditions specified in (Supporting Information 1; Table S1 and S2), in other 40 individuals of *F. microphylla*, 80 from *F. thymifolia* and 32 intermediate individuals from Garnica (see Table 1). Locus Fus 33 only amplified for *F. microphylla* and intermediate individuals, therefore it could not be included in the microsatellites analyses.

### Nuclear and chloroplast DNA sequences

Genomic DNA was chosen from 3 to 4 individuals per population, plus the 32 hybrids from the Garnica population. Chloroplast DNA (cpDNA) was amplified with the trnL-c and trnL-d primers (Taberlet *et al.* 1991), and for the cpDNA, rpL16 intron we used the primers F: 71 and R: 1661 (Kelchner and Clark 1997). The nuclear rDNA (ITS) region was amplified with the primers ITS-p4: R and ITS-p5 F (Cheng *et al.* 2016; see Supporting Information: Table S3).

All PCR reactions had a total volume of 50 μL, containing 5 μL of diluted DNA (~40 ng), 0.2 μM of each primer, and 25 μL 1x Qiagen Multiplex PCR Master Mix. In the case of trnL and ITS reactions 2.5 μL of dimethyl sulfoxide (DMSO) were added. The PCR products were examined via electrophoresis in 1.5% agarose gels and with automated capillary electrophoresis with QIAxel (QIAGEN). The PCR products were purified using purification pearls (AMPure XP, Beckman Coulter). Finally, samples were sequenced in both directions on an ABI 3720xl System (Macrogen Korea).

## Genetic Analyses

### Nuclear microsatellites

To detect genotyping errors caused by null alleles, stuttering, or large allele dropout we used Micro-checker 2.2.3 (Van Oosterhout *et al.* 2004). We looked for private alleles of each species to determine whether the hybrids possess private alleles of each parental species.

We evaluated genetic structure in the sampled populations with a principal coordinates analysis (PCoA) in GENALEX 6.5 (Peakall and Smouse 2012). STRUCTURE 2.3.4 (Pritchard *et al.* 2000) was used to calculate an admixture coefficient (*q*) for every individual. In the runs, the admixture model was employed with allele frequencies correlated, with *K* values from 1 to 10, with 10 runs for each K. In each run, a burn-in of 500 000 iterations followed by 1 000 000 Markov chain Monte Carlo (MCMC) iterations were used and then STRUCTURE HARVESTER (Earl and vonHoldt 2012) was used to determine the most likely *K* value by measuring the Δ*K*. Individuals with *q* ≥ 0.90 were considered as belonging to *F. microphylla*, while individuals with *q* ≤ 0.1 were considered of *F. thymifolia*. Individuals with 0.1 ≤ *q* ≤ 0.9 were classified as hybrids. Given the low number of private alleles for *F. thymifolia*, if we only consider the admixture coefficient (*q*) we could misclassify most hybrids as pure *F. microphylla* individuals. Therefore, the private alleles and the homozygous fixed alleles of *F. thymifolia* in Fus 31 and Fus 34 were key to identify hybrids. We used NewHybrids version 1.1 beta (Anderson and Thompson 2002), to estimate the posterior probability of each individual belonging to categories such as parental purebreds, F_1_, F_2_ and backcross, assuming that samples are composed of pure parental species and hybrids. We used the default genotype categories for first and second generations of crossing and ran 100 000 MCM with burn in a period of 50 000 with Jeffrey-type prior. Also, the allopatric populations were used as reference samples of pure individuals. The individuals were assigned to one of the six genotypic classes if *P* ≥ 0.90.

Finally, GenAlEx 6.501 (Peakall and Smouse, 2012) was used to estimate the number of alleles (*A*), number of private alleles (*A*_p_), observed (*H*_o_), and expected (*H*_e_) heterozygosity, departure from Hardy–Weinberg equilibrium, inbreeding coefficient (*F*_is_) and pairwise *F*_st_ among the parental taxa and the hybrids, and between all the populations with 999 permutations.

### Chloroplast and nuclear sequences

In total, 60 individuals were sequenced, except for the ITS ith only 38 individuals amplified despite repeated attempts. All sequences from each locus were assembled and inspected using Sequencher 4.1.4 and Phyde 0.9971 (2010) and then were aligned with Muscle 3.8.31 (Edgar 2004). Adjacent multiple base gaps were treated as a single indel because a single deletion event is the most parsimonious explanation for contiguous alignment gaps (Garrick *et al*. 2004). The trnL and rpL16 sequences were concatenated and then haplotype networks were constructed separately for chloroplast and nuclear sequences using statistical Parsimony in TCS v1.21 (Clement *et al.* 2000). ITS sequences were reconstructed with PHASE in DnaSPv.5.10 (Librado and Rozas 2009). We also calculated the number of haplotypes (*h*), haplotype diversity (*H*_d_), and nucleotide diversity (*π*), for *F. microphylla*, *F. thymifolia* and hybrids using DnaSP v.5.10 (Librado and Rozas 2009) always considering gaps as the fifth state and excluding gaps only in pairwise comparison.

### Geometric morphometry

The leaves and flowers collected for geometric morphometry were taken from a sympatric population (Garnica) and an allopatric population for each species (FmDL and FtT, see Table 1). Between 1–3 flowers and between 3–4 leaves from 10 plants per morph (female and hermaphrodite) per population were collected and then photographed with a reference scale in different positions to visualize the floral tube, the corolla, the calyx, and the leaves obtaining a total of 377, 391, 394 and 433 photographs, respectively. A different configuration of landmarks was constructed for each set of photographs to adequately represent each structure. Only one petal or sepal was used from each flower (see Supporting Information 2 and Fig. S1 for description and example of landmarks positions).

For all structures, we built a tps file from images with tpsUtil 1.33 (Rohlf 2004) and then the landmark configurations were digitized using the program tpsDig 2.31 (Rohlf 2017).

A generalized procrustes analysis (GPA) was performed for each landmark configuration of each morphological structure with the *geomorph* 4.0 package of the RStudio software 1.4.1106 (R Studio Team 2018). The GPA consists of minimizing the sum of squared distances between corresponding landmarks to extract shape data by removing information on size, location, and orientation (Klingenberg *et al.* 2012; Savriama 2018). The landmarks configuration from flowers and leaves of the same individual were averaged after superimposition, and then a GPA was done. To maximize the differences between groups relative to the variation within groups, a canonical variates analysis (CVA) was performed independently using the procrustes coordinates of leaves, floral tubes, petals, and sepals (Klingenberg *et al.* 2012). The statistical significance of pairwise differences in mean shapes was assessed with permutation tests using Mahalanobis distance (10 000 permutations per test).

## Results

### Nuclear microsatellites (nSSRs)

After exhaustive amplification tests, we were able to amplify six nuclear microsatellite loci (Fus 27, Fus 52, Fus 31, Fus 47, Fus 29, and Fus 34) in both parental species and in the 32 putative hybrids. Micro-checker 2.2.3 (Van Oosterhout *et al.* 2004) suggested possible null alleles in three populations for Fus 34 (FmDL, FmA, and HG), and in FtT, FtG, and FmG for Fus 52, Fus 31, and Fus 47, respectively. Finally, Fus 52 could have probable errors due to stuttering in FtT. In total, we found 86 alleles, and 29 (34%) are shared among *F. microphylla* and *F. thymifolia* while 39 (45%) and 17 (20%) are private alleles, respectively. Almost all hybrids had at least one private allele from each of the parental species, as evidence of their hybrid origin except for five intermediate individuals, who only had private alleles from *F. microphylla* (HG4 and HG25) or from *F. thymifolia* (HG23, HG29, and HG30). Specifically, hybrids showed four private alleles from *F. thymifolia:* one in the Fus 52 and Fus 31 loci, and two in the Fus 47 locus. In contrast, hybrid individuals shared 14 private alleles with *F. microphylla* at all the loci except Fus 29. All the individuals of *F. thymifolia*, except three (FtA7, FtG9, FtG16), are homozygous at the Fus 31 and Fus 34 loci, with the alleles 131 and 124, respectively. For *F. microphylla*, four individuals (FmDL11, FmDL30, FmA1, and FmA8) have one 124 allele, while the FmDL10, FmDL19, and FmDL26 individuals are homozygous for this allele.

**Figure 2.**
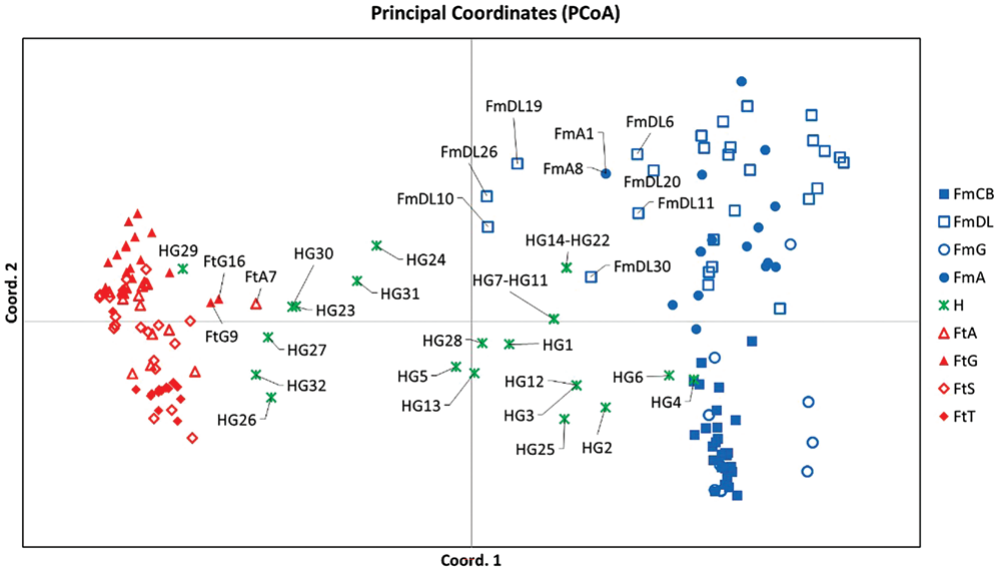
Principal coordinates analyses (PCoA) using microsatellite data of *F. microphylla*, *F. thymifolia* and their hybrids from different populations. A clear separation of the parental species and the intermediate position of the putative hybrids from the Garnica population is shown.

The PCoA clearly separated the individuals of *F. microphylla* from those of *F. thymifolia* and placed the putative hybrids in an intermediate position, although with more variation (Fig. 2). The first axis accounted for 25% of the genetic variance and separated the two parental species, while the second axis explained 9% and mainly separated the *F. microphylla* populations in two groups, one including FmDL-FmA and the other FmCB-FmG. Interestingly, the 32 putative hybrids were represented by only 19 points, and individuals (Fig. 2) HG4 and HG6 grouped with *F. microphylla* individuals; while HG29 grouped with *F. thymifolia*. Furthermore, six individuals from FmDL population (FmDL6, FmDL10, FmDL11, FmDL19, FmDL20, FmDL30) and two from FmA (FmA1, FmA8) grouped with the putative hybrids from Garnica, as well as three *F. thymifolia* individuals (FtA7, FtG9, FtG16) from the sympatric populations (Fig. 2).

**Figure 3.**
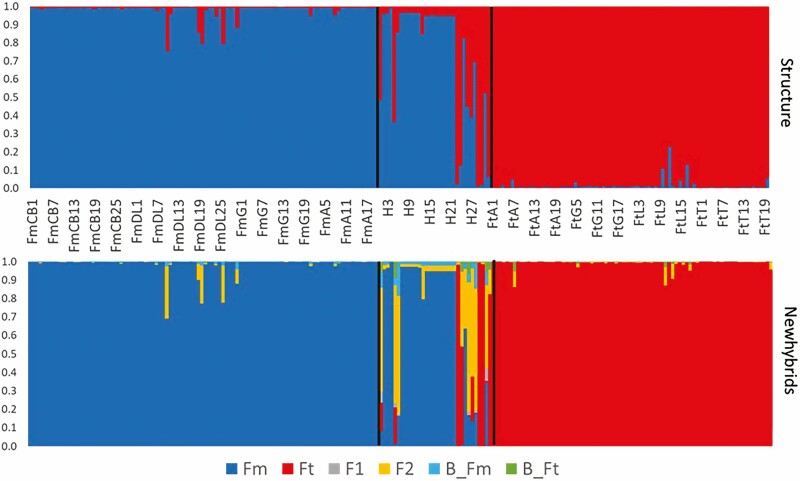
Bayesian assignment (*K* = 2) with structure for all the individuals of *F. microphylla* (blue), *F. thymifolia* (red) and the putative hybrids from Garnica population (between black bars); each vertical line represented one individual. The posterior probability for each individual was calculated with NewHybrids which indicates the possibility of belonging to the following categories: Pure species, F_1_, F_2_ and backcrosses. For both analyzes the threshold value was ≥0.90.

STRUCTURE identified two main genetic clusters, one corresponding to *F. microphylla* and the other to *F. thymifolia*. Ten out of 32 putative hybrids had an admixture coefficient between (0.1 ≤ *q* ≤ 0.90) corresponding to the hybrid category. This analysis also identified 18 hybrids as pure Fm individuals and only four as Ft pure individuals. With such a threshold value, five individuals from the allopatric population FmDL, and three from FtL had an admixture coefficient corresponding to the hybrid category. In contrast, according to the NewHybrids program the putative hybrids were classified into 18 pure Fm individuals, three pure Ft individuals, and 11 hybrids that could not be assigned to any of the six categories because they had a posterior probability between 0.45 and 0.82 (Fig. 3). In addition, the NewHybrids classification, rejected the FtA7, FtS10 and four individuals from FmDL, as pure individuals.

Genetic diversity was higher in *F. microphylla* than in *F. thymifolia* across all populations and parameters (Table 2). Even though the hybrids of Garnica only had one private allele, they showed greater genetic diversity than all the populations of *F. thymifolia* and some of the *F. microphylla* populations. Genetic differentiation was significant between all pairs of *F. microphylla*, *F. thymifolia* and hybrid populations, with *F*_*st*_ values ranging from 0.086 to 0.497 (Table 3). With respect to pairwise differentiation between parental species and hybrids, the highest *F*_*st*_ corresponds to *F. microphylla*-*F. thymifolia* comparison (0.299, *P* < 0.001), followed by Ft-HG (0.208, *P* < 0.001), and finally lower genetic differentiation was observed for Fm-HG (0.105, *P* < 0.001).

**Table 2.** Genetic diversity parameters (means ± 1 SEM) for the studied populations of *F. microphylla* and *F. thymifolia*. Fm: *F. microphylla*, Ft: *F. thymifolia*; *N:* number of analyzed individuals; *A:* average number of alleles; *A*_p_: number of private alleles; *H*_o_: average observed heterozygosity; *H*_e_: average expected heterozygosity; HWE: loci in Hardy–Weinberg equilibrium; Fis: inbreeding coefficient.

Population	*N*	*A*	*A*_p_	*H*_o_	*H*_e_	HWE	Fis
*FmCB*	30	5.67 ± 1.76	39	0.75 ± 0.08	0.63 ± 0.06	3	−0.25 ± 0.2
*FmDL*	30	6 ± 1.67		0.59 ± 0.14	0.58 ± 0.1	3	−0.04 ± 0.16
*FmG*	20	5 ± 1.37		0.73 ± 0.18	0.60 ± 0.07	6	−0.23 ± 0.19
*FmA*	20	5.33 ± 1.56		0.57 ± 0.12	0.55 ± 0.1	2	−0.09 ± 0.19
*HG*	32	6.33 ± 1.48	1	0.66 ± 0.14	0.65 ± 0.08	5	−0.05 ± 0.17
*FtA*	20	3.33 ± 1.02	17	0.32 ± 0.14	0.30 ± 0.13	1	−0.06 ± 0.03
*FtG*	20	2.83 ± 1.08		0.21 ± 0.12	0.25 ± 0.13	1	0.09 ± 0.09
*FtL*	20	4.5 ± 1.95		0.23 ± 0.13	0.26 ± 0.14	2	0.28 ± 0.2
*FtT*	20	3.67 ± 1.41		0.45 ± 0.19	0.39 ± 0.15	3	−0.06 ± 0.28

**Table 3.** Measures of genetic differentiation (*Fst*) among populations of *Fuchsia microphylla* (Fm), *F. thymifolia* (Ft) and putative hybrids (H). All *Fst* estimates were *P* < 0.001. See Table 1 for the acronyms used for each population.

	FmCB	FmDL	FmG	FmA	HG	FtA	FtG	FtL
FmDL	0.209							
FmG	0.212	0.244						
FmA	0.217	0.086	0.236					
HG	0.190	0.165	0.173	0.190				
FtA	0.435	0.421	0.477	0.461	0.276			
FtG	0.433	0.399	0.497	0.445	0.212	0.383		
FtL	0.399	0.407	0.458	0.429	0.243	0.330	0.196	
FtT	0.367	0.394	0.418	0.413	0.233	0.311	0.259	0.195

### Chloroplast sequences

**Figure 4.**
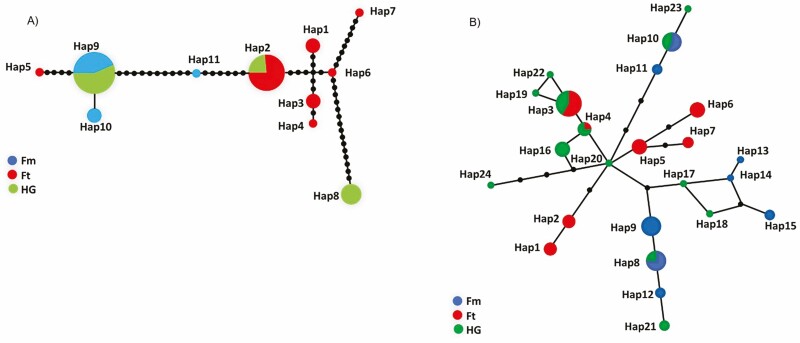
Haplotype network constructed from (A) concatenated chloroplast sequences and (B) ITS nuclear sequences with *F. microphylla* (blue), *F. thymifolia* (red), and hybrids in green. Branches represent mutations, the filled circles represent inferred unsampled or extinct haplotypes, and the frequency of each haplotype is represented by the size of the circle.

We found 11 haplotypes on the concatenated cpDNA sequences (1575 bp) with 43 variable sites including polymorphic/indel/missing sites after a codification to adjacent multiple base gaps. The software TCS with a 95% parsimony limit (connection limit = 17) produced one haplotype network (Fig. 4a). For *F. microphylla,* only three haplotypes were present, Hap9 the most common and shared with some hybrids. Hap10 was exclusive to FmCB individuals and Hap11 only was found in FmG15 individuals. The other seven haplotypes were found in *F. thymifolia* individuals. Hap2 was the most common and the only haplotype shared between some hybrids and individuals of the FtG population. The allopatric populations FtL and FtT had one (Hap1) and two (Hap3 and Hap4) exclusive haplotypes, respectively. Each one of the three FtA individuals had a different haplotype (Hap5, Hap6, Hap7). The haplotype diversity for chloroplast sequences by taxa ranged from 0.47 (Fm) to 0.86 (Ft) and the nucleotide diversity ranged from 0.001 (Fm) to 0.007 (HG; Table 5).

Although FmG15 was initially collected as a ‘pure individual’ of *F. microphylla*, it shows a unique haplotype that is much closer to *F. thymifolia*. In contrast, FtA3 has a unique haplotype more similar to *F. microphylla* than to *F. thymifolia*, and both cases indicated their hybrid origin.

### Nuclear sequences

We found 24 haplotypes on ITS sequences (625 pb) with 20 variable sites from 38 individuals (Fig. 4b). The TCS software with a 95% parsimony limit (connection limit = 10) produced one haplotype network from the reconstructed sequences (76 in total). Eight haplotypes were found in *F. microphylla* individuals but only Hap8 and Hap10 were shared with one (HG02) and two (HG23, HG30) of the putative hybrids, respectively. The allopatric population of FmCB had one private haplotype (Hap11), while the sympatric population of FmG had four (Hap12-15), and FmDL shared the Hap9 with one individual from FmA. In *F. thymifolia* populations, we found seven haplotypes and three were exclusive to FtL and FtT (Hap5, Hap6, and Hap7); the Hap1 and Hap2 correspond to the individuals of the sympatric population of FtA. In Garnica, the FtG-HG individuals shared Hap3 and Hap4. Specifically, the hybrids had nine exclusive haplotypes, four very similar to Fm (Hap17, Hap18, Hap21, and Hap23) and the other five to Ft (Hap16, Hap19, Hap20, Hap22, and Hap24; Fig. 4b). However, only 14 hybrids could be sequenced for ITS (Table 4). The ITS haplotypes observed in four hybrids were more closely related to *F. microphylla*; seven hybrids showed an opposite pattern and only the hybrids HG23 and HG32 had haplotypes mixed from the two parents. Unfortunately, we were not able to obtain data for some putative hybrids. The haplotype diversity for ITS sequences (Table 5) by taxa ranged from 0.84 (Fm) to 0.92 (HG) and the nucleotide diversity ranged from 0.0040 (Ft) to 0.0057 (HG; Table 5).

**Table 4.** Classification of hybrid plants found in Garnica population. Structure (*q*) shows the genetic proportion Fm/Ft. The NewHybrids show the genetic class assigned and its posterior probability. Chloroplast and ITS showed the haplotype(s) for each hybrid and the species to which each haplotype belongs is indicated in parenthesis. Fm = *F. microphylla*, Ft = *F. thymifolia*, HG = hybrids from Garnica.

Hybrid	Structure (*q*)	NewHybrids	Chloroplast	ITS
HG1	0.487/0.513	F_2_ (0.562)	Hap9 (Fm)	NS
HG2	0.958/0.042	Fm (0.96)	Hap9 (Fm)	Hap8 (Fm)
(HG3, HG12)	0.963/0.037	Fm (0.96)	Hap9 (Fm)	Hap17,18 (HG)
HG4*	0.992/0.008	Fm (0.99)	Hap9 (Fm)	NS
HG5	0.367/0.633	F_2_ (0.64)	Hap2 (Ft)	NS
HG6	0.858/0.142	F_2_ (0.64)	Hap2 (Ft)	NS
(HG7, HG8, HG9, HG10, HG11)	0.966/0.034	Fm (0.97)	Hap9 (Fm)	Hap3 (Ft), Hap4 (Ft), Hap16 (HG)
HG13	0.847/0.153	Fm (0.80)	Hap2 (Ft)	Hap16 (HG), Hap19 (HG)
HG14, HG15,				
HG16, HG17,	0.949/0.051	Fm (0.95)	Hap2 (Ft)	NS
HG18, HG19,				
HG20, HG21,				
HG22				
HG23*	0.02/0.98	Ft (0.98)	Hap8 (HG)	Hap10(Fm)
HG24	0.124/0.876	Ft (0.53)	Hap8 (HG)	NS
HG25*	0.825/0.175	Fm (0.63)	Hap8 (HG)	NS
HG26	0.448/0.552	F_2_ (0.70)	Hap9 (Fm)	Hap20(HG), Hap21(HG)
HG27	0.389/0.611	F_2_ (0.59)	Hap9 (Fm)	NS
HG28	0.695/0.305	F_2_ (0.66)	Hap8 (HG)	NS
HG29*	0.01/0.99	Ft (0.99)	Hap8 (HG)	Hap4(Ft), Hap22(HG)
HG30*	0.02/0.98	Ft (0.98)	Hap8 (HG)	Hap10(Fm), Hap23(HG)
HG31	0.52/0.48	F_2_ (0.45)	Hap2 (Ft)	NS
HG32	0.064/0.936	Ft (0.82)	Hap9 (Fm)	Hap21(HG), Hap24(HG)

The hybrids in parentheses have the same SSR genotype and the same admixture coefficients and posterior probabilities. NS = sequences could not be obtained. *Only have private alleles from one parental species.

**Table 5.** Genetic diversity in *F. microphylla, F. thymifolia* and putative hybrids. *N*: number of individuals analyzed for chloroplast and ITS respectively; *h*: number of haplotypes; *H*_d:_ haplotype diversity; *π*: nucleotide diversity.

Loci	Chloroplast (trnL-rpL16)			ITS		
Species	*h*	*H*_d_	*π*	*h*	*H*_d_	*π*
Fm (*N* = 15, 13)	3	0.4725	0.0011	8	0.840	0.0048
Ft (*N* = 14, 12)	7	0.8681	0.0039	7	0.855	0.0040
HG (*N* = 32, 13)	3	0.655	0.0073	13	0.926	0.0057
Total (*N* = 60, 38)	11	0.7667	0.0070	24	0.938	0.0059

GenBank accession numbers: (a) trnL sequences: OR778498-OR778557; (b) rpL16: OR901332-OR901391, and (c) ITS: OR853053-OR853090.

### Geometric morphometrics

**Figure 5.**
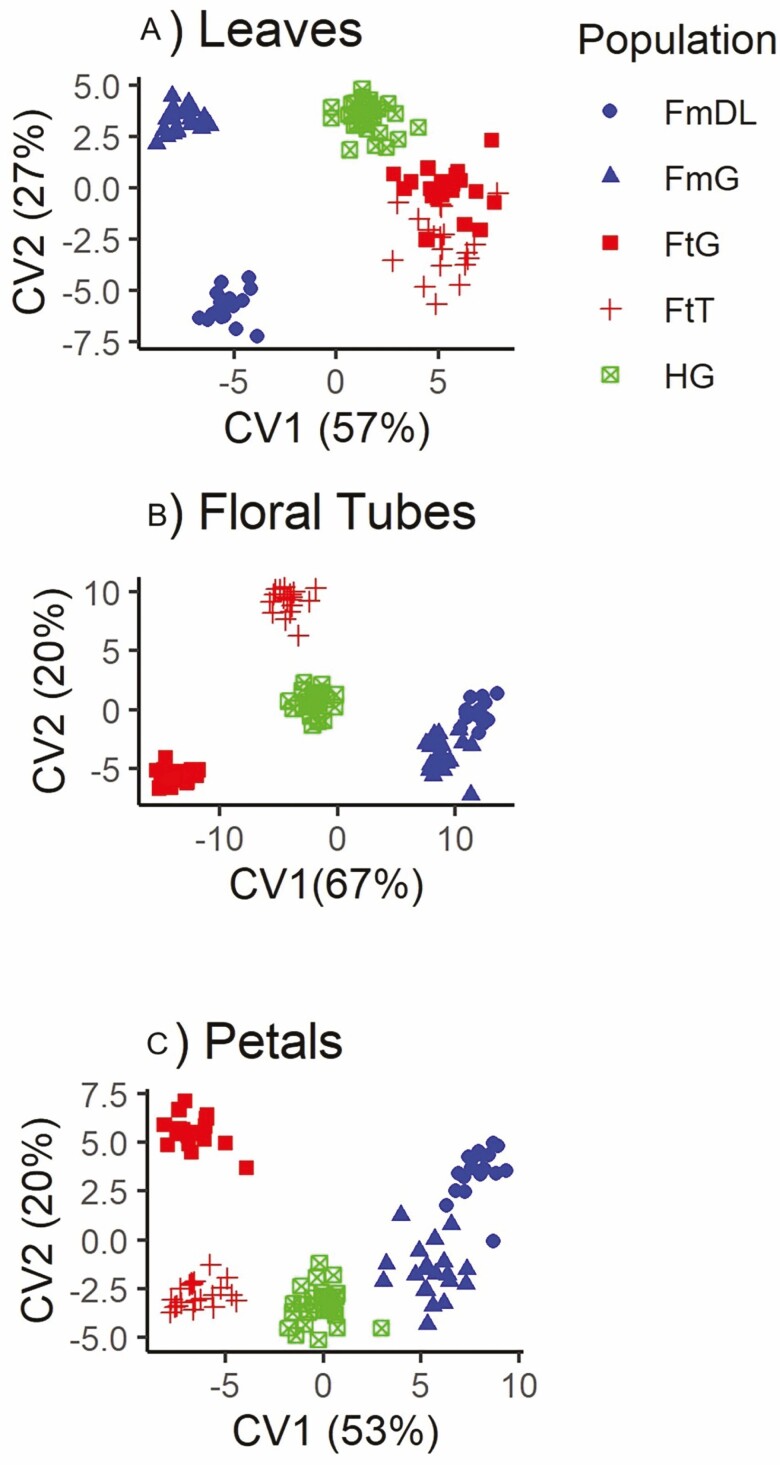
Canonical variates analyses (CVA) of (A) leaves, (B) floral tubes, and (C) petals of individuals from different populations of *F. microphylla* (blue), *F. thymifolia* (red), and hybrids from Garnica (green).

According to CVAs, the leaves, floral tubes, and petals showed a clear morphometric distinction between the two *Fuchsia* species and the putative hybrids (Fig. 5). Populations within *F. thymifolia* only showed moderate overlap in the shape of the leaves. In contrast, the CVA of floral structures displayed a greater intraspecific separation in shape. For *F. microphylla* populations the shape of the floral tubes and petals were more uniform, while leaves and petals showed more separation between populations. Mahalanobis distances range from 6.74 (FtG vs FtT) to 14.32 (FtT vs FmG) for leaves, 9.2 (FmG vs FmDL) to 26.5 (FtG vs FmDL) for floral tubes, and 9.73 (FmG vs FmDL) to 16.18 (FtT vs FmDL) for petals, and all permutation tests indicated that mean shapes differ significantly among populations (*P* < 0.01 in pairwise permutation tests between populations).

## Discussion

The results of the present study provided the first strong evidence of natural hybridization in *Fuchsia,* supported by analyses of floral and leaf morphology, and genetic markers. The results of this study support the previously pointed-out weakness of the reproductive barriers in this genus, based on a large number of artificial hybrids between other *Fuchsia* species (Berry *et al.* 2004; Talluri 2012).

### Hybrid identification with molecular markers

The different molecular markers used in this study confirmed that several intermediate individuals are the result of hybridization between *F. microphylla* and *F. thymifolia*. The nuclear microsatellites allowed us to find private alleles of both species in most hybrids. In addition, the SSRs confirmed a high genetic differentiation between *F. microphylla* and *F. thymifolia*, and showed that backcrosses to both species are occurring. Microsatellites also reduced the 32 initial putative hybrids to only 19 different genotypes, and from those, structure identified nine as purebread individuals, five from *F. microphylla* and four from *F. thymifolia* and the remaining hybrids had different degrees of genetic admixture (Fig. 3). In addition, according to NewHybrids using a threshold value of 0.90, eight putative hybrids were classified as pure individuals, but the program could not assign the rest of the putative hybrids from Garnica to the F_1_ hybrid category, so they could be considered as hybrids of later generations. In other hybrid systems, the threshold values used for NewHybrids are decided under two main criteria: (i) the threshold value is applied to each category (pure species, F_1_ hybrids, backcrosses) separately, by assigning only the individuals with *q* ≥ T*q* and leaving the other individuals unassigned or, (ii) a threshold value above 0.9 or 0.94 is used for pure individuals and a lower threshold for hybrid categories that may range from 0.5 to 0.80 (Zha et *al*. 2008; Burgarella et *al.* 2009; McIntosh et *al*. 2013; Tsy et *al.* 2013; Vega et *al*. 2013). The loss or gain of the homozygous condition on Fus 31 and Fus 34, and the results found in the PCoA, the structure and the NewHybrids analysis indicated the presence of other individuals from the allopatric populations with genetic admixture in FmDL, FtA, and FtL populations. In contrast, individuals from sympatric populations initially collected as ‘pure’ but with a certain degree of genetic admixture could suggest introgressive hybridization as in other hybridizing genera as *Silene* (Minder *et al.* 2007), *Quercus* (Lee *et al.* 2014) and *Vincentoxicum* (Li *et al.* 2016). This scenario may happen when hybrids backcross more frequently with one parental species, providing a greater genomic contribution in the hybrids, which may be confused as pure individuals, as in the case of hybrids found in the sympatric populations of FtA and FtG. On the other hand, hybrids identified in the allopatric populations might be indicating past events of introgressive hybridization, or incomplete lineage sorting, but distinguishing between them is a difficult task. Although hybridization is expected to leave traces only under sympatric conditions, incomplete lineage sorting leads to a spread-out pattern of shared genetic variation (Goetze *et al.* 2017).

In angiosperms, the DNA of chloroplast is generally maternally inherited, potentially allowing to identify the maternal progenitor of hybrids (Burgess *et al.* 2005; Zhou *et al.* 2008; Yuan *et al.* 2010; Matos et *al.* 2016). The hybrids of the Garnica population share chloroplast haplotypes from both species, meaning that hybridization may occur in both directions. Specifically, from the 19 hybrid genotypes eight have *F. microphylla* haplotypes, five have *F. thymifolia* haplotypes, and six have new haplotypes closer to *F. thymifolia* (Table 4). The individual FmG15 has a unique haplotype very similar to the Hap2 of FtG, even though it was assigned as pure *F. microphylla* according to the *q-*value. In turn, FtA3 shows a unique haplotype very similar to the Hap9 of *F. microphylla* but with a *q-*value of *F. thymifolia*.

Furthermore, only the allopatric populations FmCB, FtL, and FtT showed private haplotypes, contrasting to the allopatric FmDL population which shares haplotypes with the two sympatric populations of *F. microphylla*. This could reinforce the hypothesis that the population of Desierto de los Leones has undergone more recent introgression compared to the other allopatric populations.

For biparentally inherited markers such as the ITS region, we expected hybrids to have both maternal and paternal genetic components as well as their own copies resulting from genetic recombination (Matos *et al.* 2016). The ITS region is a universal species-specific marker for plants and fungi, typically having several hundred copies within plant genomes, meaning that the signature of autopolyploidization, recent hybridization, or introgression, might be detected unless enough generations have passed for concerted evolution to homogenize all ITS copies within the genome (Nazre 2014; Wan *et al.* 2014). The ITS region has been used to detect hybrids in many different hybridizing species (Fuertes-Aguilar *et al.* 1999; Hardig *et al.* 2000; Tsukaya *et al.* 2003; Wu *et al.* 2009; Korpelainen *et al.* 2010; Nazre 2014). In *Fuchsia*, individuals from allopatric populations of both species exhibited only one allele at the ITS region, while some pure individuals from sympatric populations and most hybrids were heterozygous, confirming their hybrid origin. For example, the hybrids HG7 to HG11 shared one allele with *F. thymifolia* individuals from Garnica and the other allele was very similar to *F. thymifolia*. By contrast, HG12 hybrid showed two different alleles very similar to individual FmG15 previously identified as pure *F. microphylla* but with cpDNA haplotype more similar to *F. thymifolia*.

The different molecular markers indicated that there are different classes of hybrids within the sympatric population of Garnica. However, many intermediate individuals from Garnica previously classified as pure individuals, show chloroplast or nuclear haplotypes that do not correspond to the species they were classified to. Thanks to this variety, the hybrid lineage has been able to persist within the population, since surely some genotypes have been able to reproduce successfully, functioning as a bridge for gene flow between both parental species.

### Morphological identification

Hybrids have been shown to display a mosaic of intermediate, parental, and transgressive characters, rather than just being intermediate (Rieseberg *et al.* 1993). Traditionally, morphometric comparisons between populations were based on the analysis of differences in their linear dimensions (Toro *et al.* 2010), but new methods that generate quantitative descriptions of shapes are quite useful for comparing shapes within- and among-species (Jensen *et al.* 2002). Geometric morphometric analyses have been applied to discriminate between parental species and their hybrids in plants, mainly using leaf shape (Jensen *et al.* 2002; Lexer *et al.* 2009; Viscosi *et al.* 2009; Peñaloza-Ramírez *et al.* 2010; Liu *et al.* 2018) and to a lesser degree flower shape (Shipunov and Bateman 2005; McCarthy *et al.* 2016; Pisano et *al*. 2018). In this study, in addition to leaves, we included various floral structures and found that the shape of leaves and the floral tube differentiated the two species to a greater extent, however, *F. microphylla* has less intraspecific variation in floral tube shape while *F. thymifolia* has less intraspecific variation in leaf shape. The putative hybrids have leaves more similar to those of *F. thymifolia* and flower tubes are intermediate between their parents.

Although the petals and sepals also allowed to differentiate both species, these structures showed greater variation in their shape, and the putative hybrids had petals and sepals more similar to those of *F. microphylla*. In addition, in the sympatric population of Garnica it is easy to recognize both parental species, because *F. microphylla* is a small shrub (less than 1 m) with purplish flowers, while *F. thymifolia* may reach 2–3 m and their flowers change with age from white to purplish (Cervantes and Cuevas 2023). The different hybrids found show different flower colors as well as different reproductive fitness (*C. Cervantes*, unpubl. data).

### Does gynodioecy facilitate hybridization?

*Fuchsia microphylla* and *F. thymifolia*, are two morphologically gynodioecious but functionally dioecious species and both are visited by *Bombus eppiphiatus* (Cuevas *et al.* 2014; Cervantes *et al.* 2018). Although the degree of overlap in flowering time varies every year, hermaphrodite plants of *F. thymifolia* begin to flower earlier than female plants. In *F. microphylla* the opposite pattern is observed; female plants blooms earlier than hermaphrodites (Cinthya Cervantes, unpubl. data). This pattern suggests that the direction of hybridization could be from *F. thymifolia* as a pollen donor to *F. microphylla* as a recipient; however, the genotypes found in the hybrids indicated a symmetric bidirectional hybridization. Some hybrids of later generations were misidentified as individuals belonging to the parental species, two of them with the maternal progenitor of *F. thymifolia*. Therefore, other factors might be promoting bidirectional hybridization. For example, the little or no nectar production in the hermaphrodite flowers of *F. thymifolia* (Cervantes *et al.* 2018) could encourage pollinators to visit these flowers in search of pollen and later, when searching for nectar, they probably visit the female flowers of both species as well as the hermaphrodite flowers of *F. microphylla*.

Finally, clonal reproduction and possible apomixis occur in *F. microphylla* and *F. thymifolia* (Cuevas *et al*. 2014; Cervantes *et al*. 2018; Cervantes and Cuevas 2023) and it might be present in hybrids (Cinthya Cervantes, pers comm.). For example, hybrids that showed identical genotypes and haplotypes were growing near each other. In the Garnica population, these phenomena may help to maintain the hybrid genotypes until they can reproduce successfully. Furthermore, it will be important to assess the reproductive biology of hybrids because most hybrids in Garnica are female individuals.

## Conclusion

Natural hybridization between *F. microphylla* and *F. thymifolia* was confirmed with molecular and morphometric studies within the sympatric population of Garnica, and it seems that most of the hybrids are from later generations and that hybridization is bidirectional. Future phylogeographic analysis of both species will be important to understand more the hybridization between these closer species of *Fuchsia*.

## Supporting Information

The following additional information is available in the online version of this article –

**Supplementary Note 1**Microsatellite development protocol and characterization of 15 microsatellite loci from *F. microphylla* (Onagracea).

**Supplementary Note 2** Description of landmarks position for each flower structure and leaves.

**Table S1.** Characteristics of microsatellite loci developed for *Fuchsia microphylla*

**Table S2.** Genetic diversity parameters for two populations of *Fuchsia microphylla. N:* number of individuals; *N*_*a*_: number of alleles; *H*_*o*_: observed heterozygosity; *H*_*e*_: expected heterozygosity; HWE: Hardy–Weinberg equilibrium; ns: no significant, * *P* < 0.05, ** *P* < 0.01, ***P < 0.001.

**Table S3.** Name and sequences of the forward and reverse primers of each of the markers used. Description of the PCR conditions for each reaction and references to the primers.

**Figure S1**. Example of landmarks position in (a) petals, (b) floral tube, and (c) leaf.

plad089_suppl_Supplementary_Figures_1-2_Tables_S1-S3

## Data Availability

The data underlying this article are available in the article and in its online Supporting Information.
