# Supplementary material for: Molecular and morphological evidence of hybridization between two dimorphic sympatric species of Fuchsia (Onagraceae)
Source: AoB Plants. 2023 Dec 21;16(1):plad089. doi: 10.1093/aobpla/plad089 (PMC10783250; doi:10.1093/aobpla/plad089)

Supporting Information 1 (SI1): Microsatellite development protocol and characterization of 15 microsatellite loci from *F. microphylla* (Onagracea).

Genomic DNA was extracted using the DNeasy Plant Mini Kit (QIAGEN, Valencia, California, USA) from a single *F. microphylla* individual collected from the population Desierto de los Leones (FmDL). DNA was then sent to the microsatellite-development company, Genetic Marker Services (Brighton, UK; www.geneticmarkerservices.com) to isolate polymorphic loci.

The microsatellite isolation was based on the production of an enriched library, using hybridization capture protocol. Enrichment involved incubating adaptor-ligated, restricted DNA, with filter-bonded synthetic repeat motifs: [AG]17, [AC]17, [AAC]10, [CCG]10, [CTG]10, and [AAT]10. The filters were then washed at a range of temperatures (50-60°C) and SSC (saline-sodium citrate) stringencies (0.5X-2X) to optimize enrichment. Later 41 microsatellite-positive *Escherichia coli* clones were detected and sequenced, from which 19 contained repeat motifs with sufficient flanking regions to design forward/reverse primers pairs. Primers were designed for the most promising loci using the online primer design software PRIMER 3.0 (Rozen and Skaletsky, 2000). A search for primers pairs that amplifying product from 100-250 bp was performed, to help minimize later multi-loading overlap ambiguities during sequencer genotyping. The primers were then tested on eight individuals, using a touchdown PCR protocol. PCR amplifications were performed in a 25 µL final volume containing 7 pmol of each primer, 1.5 mM of $MgCl_2$, 0.2 mM of each dNTP, 1 X PCR buffer, 0.8µg/µL BSA, 0.5 U Taq Polymerase, and 1.5 µL of DNA diluted 20-fold. Touchdown PCR consisted of 32 cycles of denaturation at 95°C for 60 s, annealing for 60 s and 2 cycles each 64–59°C; 10 cycles 58°C; 10 cycles 57°C, elongation at 72°C for 60 s, and a final extension at 72°C for 5 min. Products were checked for specificity, active

polymorphism and null alleles, on cooled high-resolution gels, consisting of 4% MetaPhor (Lonza) agarose in TAE, run in a cold room at 10°C.

Fifteen pairs of microsatellite primers designed for *F. microphylla* were tested on 60 individuals from two different populations (FmDL and FmCB) from which DNA was extracted from leaf tissue using a Promega extraction kit (Wizard A1125). The DNA was then quantified with a bio spectrophotometer and dilutions of each sample were prepared. The 12.5- μ L PCR cocktail contained: 1 μ L of diluted DNA (~10 ng), 0.25 μ M reverse primer, 0.25 μ M forward primer (labeled with PET, FAM, NED, or VIC), and 6.25 μ L 1x Qiagen Multiplex PCR Master Mix. From the 15 primer pairs tested, two were monomorphic, six were unscorable, and seven were polymorphic. The PCR cycling program had an initial denaturation of 95°C for 15 min; 35 cycles of 94°C for 30 s, annealing temperature (Table S1) for 90 s, and 72°C for 1 min, and a final extension at 72°C for 30 min. For Fus 31 the following program was used: an initial denaturation of 95°C for 15 min; 35 cycles of 94°C for 60 s, annealing temperature of 62.5°C, and 72°C for 1 min, and a final extension at 72°C for 10 min. PCR reactions were made for each locus and then PCR products were purified and quantified. Then amplification products of 2-3 loci with different fluorophores were mixed with 0.25 μL of LIZ 600 ladder and 9 μL of Hi-Di formamide for subsequent fragment sizing on an ABI 3500 Genetic Analyzer (Applied Biosystems). Alleles were visualized and scored using Geneious version 8.1.9 (Anderson and Thompson, 2002). Number of alleles observed and expected heterozygosity and deviations from Hardy–Weinberg equilibrium were estimated using GenAlEx 6.5 (Peakall and Smouse 2012; Table S2).

Supporting Information 2 (SI2): Description of landmarks position for each flower structure and leaves.

a) In petals two landmarks were placed (one at the tip and one at the middle base) and a curve on each side with 30 semi landmarks each one (Fig. S1a).
b) In the sepals, a landmark was placed at each end of the base thereof, as well as a landmark at the tip of the sepal, and a curve on each side with 30 semi landmarks (Fig. S1b).
c) In the floral tubes six landmarks were placed, two in the intersection of the ovary with the pedicel, two at the intersection of the ovary with the floral tube and two more at the intersection between the floral tube and the sepals, as well as four curves, two over each side of the ovary with 10 semi landmarks for each one and two curves over each side of the tube with 20 semi landmarks (Fig. S1c).
d) In the axial surface of the leaves were digitized two landmarks (one at the apex and one at the base of each leaf) and one curve at each side of the leaf with 70 semi landmarks to represent the edge of leaves (Fig. S1d). All points in curves were equidistantly placed.

Table S1. Characteristics of microsatellite loci developed for *Fuchsia microphylla*.

Locus	Primer sequence (5'-3')	Repeat motif	Allele size range (bp)	*Ta* (°C)	GenBank Accession no.
**Fus 27**	F: CACTGGAAGAGAAAGGGAAA<br>R: GGTGATGAATTCTGTCTTTGC	(AG)23	122-176	60	OR786635
**Fus 29**	F: GTTACGGAATGGAAATGGTACG<br>R: CCGTTTTATCCATTTTCACAAGTC	(GT)21	154-182	55	OR786637
**Fus 31**	F: GGGAACCCGATTTCATTTCT<br>R: TCTTCTTGGGGACCTTGATG	(TGA)9	126-159	62	OR786639
**Fus 33**	F: GGGAAGACCACCATTTTCCT<br>R: ACCCGAACTTTCACCTCCTT	(GA)21	185-225	60	OR786640
**Fus 34**	F: GCCGAGTCTAACTCGTCTCC<br>R: GCATGCTTAGTGGAACTCGG	(GT)48	124-200	63	OR786641
**Fus 47**	F: TCGACCTCAATTCTCAATACC<br>R: TGAGATGGGCAAAATAAAGA	(TC)19	174-224	61	OR786647
**Fus 52**	F: CCTGCCATAACCTCTGTCATC<br>R: TTGCATTGAAATTCTTAGCTTGA	(TG)9	191-197	60	OR786649
**Fus 48**	F: TGGTTTTATGTGTGAAATGGA<br>R: ACTTAGGCGGATCACAAAAC	(TG)9	117-119	57	OR786648
**Fus 35**	F: TGGATTTTTGGATGAAGTTGC<br>R: TCACGCAATCGTCTCTCACT	(GT)13	127	60	OR786642
**Fus 43**	F: CTCCAGCGTTTGATTTCTCA<br>R: CTCGTCTTCACATCACACACA	(TG)9	140	59	OR786644
**Fus 28**	F: GCTCTCTGCCGTAGGATCTG<br>R: CCACGAAATCACCCAAAAAC	(TG)11	157	60	OR786636
**Fus 44a**	F: TAGACTCCGACGACACGAGA<br>R: AGATTTGGTTGCGGGACATT	(AC)21	194	60	OR786645
**Fus 42**	F: GCTTGTGAAGCGCTACTCTG<br>R: CATCAACAACAATCGTCACCA	(GTT)10	144	60	OR786643
**Fus 30**	F: CTTTTTGCTTGCCGCGATAC<br>R: TGTGCTTTATTTGTGCGTCTG	(AC)7	105	63	OR786638
**Fus 44b**	F: AACATGCCGTTTCATTACCG<br>R: CATGAGGCACGTAAGGCATA	(GAA)14-TAAG-(AAC)26	212	64	OR786646

Ta= annealing temperature.

Table S2. Genetic diversity parameters for two populations of *Fuchsia microphylla*. *N:* number of individuals; N_a: number of alleles; *Ho:* observed heterozygosity; H_e: expected heterozygosity; *HWE:* Hardy-Weinberg equilibrium; *ns:* no significant, * P<0.05, ** P<0.01, *** P<0.001.

	Desierto de los Leones (*N*=30)				Cerro Burro (*N*=30)			
**Locus**	N_a	H_o	H_e	***HWE***	N_a	H_o	H_e	***HWE***
**Fus 27**	10	0.73	0.77	*Ns*	11	0.87	0.75	*ns*
**Fus 52**	2	1.00	0.50	***	2	0.70	0.47	**
**Fus 31**	3	0.43	0.51	***	5	0.77	0.66	*ns*
**Fus 47**	12	0.83	0.84	*Ns*	11	0.87	0.77	**
**Fus 29**	2	0.87	0.49	***	2	0.13	0.12	*ns*
**Fus 34**	5	0.63	0.69	*Ns*	6	0.19	0.65	***
**Fus 33**	14	0.93	0.87	*Ns*	13	0.87	0.83	*

Voucher information: IEB Herbarium (Instituto de Ecología, A.C. Pátzcuaro, México) 92695-J.M. Escobedo G.

Table S3. Name and sequences of the forward and reverse primers of each of the markers used. Description of the PCR conditions for each reaction and references to the primers.

**Primers**	**Sequences**	**PCR conditions**	**Reference**
trnL-c	CGAAATCGGTAGAC GCTACG	95°C for 15 min, followed by 35 cycles of 30 s at 95°C, 30 s at 50°C and 2 min at 72°C, with a final step of 7 min at 72°C	Taberlet 1991
trnL-d	GGGGATAGAGGGAC TTGAAC		
rpL16 F:71	GCTATGCTTAGTGT GTGACTCGTTG	95°C for 15 min, followed by 35 cycles of 1 min at 95°C, 1 min at 48 or 50°C (depending on the species) and 4 min at 65°C, with a final step of 10 min at 65°C	Kelchner and Clark 1997
rpL16: R:1661	CGTACCCATATTTTT CCACCACGAC		
ITS-p4: R	CCGCTTAKTGATATGC TTAAA	95°C for 15 min, followed by 35 cycles of 4 min at 94°C, 30 s at 94 °C, 45 s at 55°C and 1 min at 72°C, with a final step of 10 min at 72°C	Cheng et al. 2016
ITS-p5: F	CCTTATCAYTTAGAGG AAGGAG		

Figure S1. Example of landmarks position in a) petals, b) floral tube, and c) leaf.

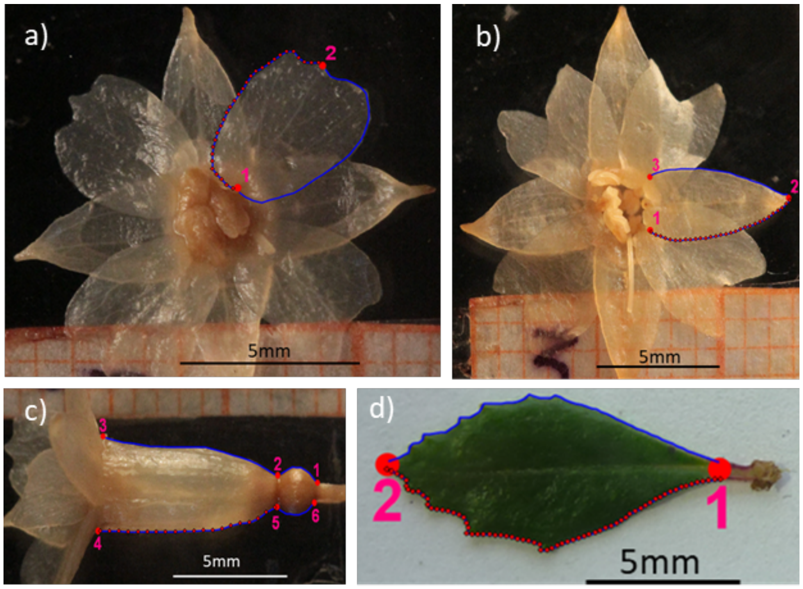

Supplement: plad089_suppl_Supplementary_Figures_1-2_Tables_S1-S3 [file plad089_suppl_supplementary_figures_1-2_tables_s1-s3.pdf]
